# The Impacts of Migraine among Outpatients with Major Depressive Disorder at a Two-Year Follow-Up

**DOI:** 10.1371/journal.pone.0128087

**Published:** 2015-05-22

**Authors:** Ching-I Hung, Chia-Yih Liu, Ching-Hui Yang, Shuu-Jiun Wang

**Affiliations:** 1 Department of Psychiatry, Chang Gung Memorial Hospital at Linkou and Chang Gung University School of Medicine, Tao-Yuan, Taiwan; 2 Department of Nursing, Chang Gung University of Science and Technology, Tao-Yuan, Taiwan; 3 Faculty of Medicine, National Yang-Ming University School of Medicine and Neurological Institute, Taipei Veterans General Hospital, Taipei, Taiwan; Institute of Psychiatry, UNITED KINGDOM

## Abstract

**Background:**

No study has investigated the impacts of migraine on depression, anxiety, and somatic symptoms and remission at the two-year follow-up point among patients with major depressive disorder (MDD). This study aimed to investigate the above issues.

**Methods:**

Psychiatric outpatients with MDD recruited at baseline were investigated at a two-year follow-up (N = 106). The Hamilton Depression Rating Scale, Hospital Anxiety and Depression Scale, and Depression and Somatic Symptoms Scale were used. Migraine was diagnosed according to the *International Classification of Headache Disorders*, 2^nd^ edition. The patients were divided into no migraine, inactive migraine, and active migraine subgroups. Multiple logistic regressions were used to investigate the significant factors related to full remission of depression.

**Results:**

Among patients without pharmacotherapy at the follow-up, patients with active migraine had significantly greater severities of anxiety and somatic symptoms as compared with patients without migraine; moreover, patients with active migraine had the lowest improvement percentage and full remission rate. There were no significant differences in depression, anxiety, and somatic symptoms between patients with inactive migraine and those without migraine. Active headache at follow-up was a significant factor related to a lower full remission rate.

**Conclusions:**

Active headache at follow-up was associated with a lower rate of full remission and more residual anxiety and somatic symptoms at follow-up among patients with migraine. Physicians should integrate a treatment plan for depression and migraine for the treatment of patients with MDD.

## Introduction

Migraine, mood disorders, and anxiety are comorbid with each other and also interact [1–11]. Among patients with migraine, comorbidity with depression and/or anxiety is related to a greater severity of headache, a poorer quality of life, more disability, and a higher risk of suicide [12–14]. At long-term follow-up, depression and anxiety predict a poorer prognosis of migraine and are related to the transformation of episodic migraine to chronic migraine [13–15].

Conversely, migraine is also common among patients with depression or major depressive disorder (MDD) [7, 10, 16, 17]. Depressive patients with migraine are associated with 1) more depressive episodes and clinical features of bipolar spectrum traits [18]; 2) greater severities of depression, anxiety, and somatic symptoms, as well as a poorer health-related quality of life (HRQoL) [16, 17, 19, 20]. Migraine is one of the important factors related to somatic symptoms among patients with depression and anxiety [19, 21]. However, most of the previous studies were cross-sectional studies. The impact of migraine on the outcome of depression has rarely been addressed [22]. To the best of our knowledge, no study has examined the role of migraine or headache on the two-year follow-up of patients with MDD. Understanding the impact of migraine or headache on depression, anxiety, and somatic symptoms among patients with MDD is important because migraine is common and preventable. Moreover, some pharmacotherapy for depression is also effective for the treatment of migraine [1, 23].

Therefore, this study aimed to investigate the impacts of migraine at baseline on depression, anxiety, and somatic symptoms and remission at a two-year follow-up among patients with MDD. We hypothesized that comorbidity with migraine at baseline might be associated with greater severities of depression, anxiety, and somatic symptoms at follow-up.

## Method

### Subjects

The study enrolled patients at baseline from January 2004 to January 2005 in the psychiatric outpatient clinic of Chang Gung Memorial Hospital, a medical center in northern Taiwan, and was approved by the Institutional Review Board of the same hospital. Participants were consecutive outpatients (18–65 years) who met the DSM-IV-TR criteria for MDD and were experiencing a current major depressive episode (MDE), confirmed by the Structured Clinical Interview for DSM-IV-text revision (TR) Axis I Disorders [24], and who had not taken antidepressants or any other psychotropic drugs within the previous 2 weeks. To prevent depressive symptoms from being confounded by other conditions, the following exclusion criteria were established: 1) a history of substance dependence or abuse without full remission in the previous month; 2) psychotic symptoms, catatonic features, or severe psychomotor retardation with obvious difficulty being interviewed; and 3) chronic medical diseases that should be regularly treated with long-term pharmacotherapy, such as hypertension, diabetes mellitus, and other medical diseases, except for headache. In the second point of the exclusion criteria, this study had excluded patients with psychotic symptoms, catatonic features, or severe psychomotor retardation, who might have a compromised capacity to consent. Therefore, the enrolled patients with MDD had the capacity to consent. A written informed consent, based on the guidelines regulated in the Declaration of Helsinki, was obtained from all subjects prior to study enrollment.

### Instruments

Three scales—the Depression and Somatic Symptoms Scale (DSSS), the Hamilton Depression Rating Scale (HAMD), and the Hospital Anxiety and Depression Scale (HADS)—were used to evaluate depression, anxiety, and somatic symptoms [25–27]. The HAMD, an interviewer-administered scale and one of the most popular scales for evaluating depression, included 17 items. The DSSS [26], a self- administered scale, includes 12 items in the Depression Subscale (DS) and 10 items in the Somatic Subscale (SS). The reliability and validity of the DSSS have been reported [28, 29]. The severity of each item for the DSSS was rated at one of four levels (0–3 scores): “Absent”, “Mild”, “Moderate”, and “Severe”. The HADS, a self- administered scale, includes 7 items for anxiety (HADS-A) and 7 for depression (HADS-D).

### Assessment of headache

At baseline, all patients completed a structured headache intake form, which was designed to meet the operational criteria of the International Classification of Headache Disorders, 2nd edition (ICHD-2) [30]. The form emphasized the collection of information needed to classify migraine and other types of headache. An experienced headache specialist at baseline, who was blind to the results of the psychiatric diagnoses and psychometric scales, interviewed all patients after they had completed the headache intake form and made headache diagnoses based on the ICHD-2.

At baseline, subjects evaluated their average headache intensity during the previous week using a visual analog scale, with 0 representing “no headache” and 10 representing “headache as severe as I can imagine.” The total number of headache days in the past month was collected. At the two-year follow-up, the same procedures were repeated.

### Procedure

During the two-year period, these patients were treated as general psychiatric outpatients without controlling their pharmacotherapy. Therefore, some patients might continue pharmacotherapy and others might drop out from pharmacotherapy based on patients’ willingness to continue treatment. Two years later, the subjects were invited to attend a follow-up. Subjects who were undergoing pharmacotherapy in the index follow-up month were categorized into the treated group and those who were not were categorized into the undertreated group. In the treated and undertreated groups, patients were further divided into three subgroups based on the degree of their headache disturbance at follow-up. Patients with migraine, diagnosed at baseline, and the headache item in the SS rated as “Absent” or “Mild” at follow-up were categorized as the inactive headache group, and those rated as “Moderate” and “Severe” at follow-up were categorized as the active headache group. A mild degree was categorized into the inactive headache group because a migraine attack often has a moderate to severe intensity and causes significant discomfort. Therefore, the MDD patients in the treated and undertreated groups were categorized as patients without migraine (no migraine subgroup), patients with migraine at baseline and inactive headache at follow-up (inactive migraine subgroup) and patients with migraine at baseline and active headache at follow-up (active migraine subgroup).

At the two-year follow-up, two kinds of full remission were investigated. First, full remission of depression at the follow-up point was defined as a HAMD score ≤ 7. Secondly, physicians clarified whether the patients had full remission for more than two months and the duration of full remission in the past two years. To understand the improvement degree of psychometric scales between the baseline and follow-up point, the improvement percentage (IP) was used, which was calculated by (score at baseline—score at follow-up) × 100% / score at baseline.

### Statistical methods

All statistical analyses were performed using SPSS for Windows 15.0. The independent *t* test, paired *t* test, and chi-square test were used when appropriate. Non-parametric models, such as the Wilcoxon test, Mann-Whitney U test, and Kruskal-Wallis H test, were used in appropriate situations when subjects were divided into no migraine, inactive migraine, and active migraine subgroups.

General linear models were used to calculate the estimated difference contributed by migraine after controlling for demographic variables. In the analysis, the no migraine subgroup was used as a reference subgroup. To identify the associations of migraine, headache at follow-up, and the outcome of MDD, multivariate logistic regression with the enter method was performed. The dependent variable was the presence of full remission (HAMD score ≤ 7) at the follow-up point. The independent variables consisted of five demographic variables at baseline (age, gender, marital status, employment, and educational years), pharmacotherapy or not at the follow-up point, HAMD score at baseline, migraine or not at baseline, and active headache or not at the two-year follow-up. In all statistical analyses, a two-tailed *p* value < 0.05 was considered statistically significant.

## Results

### Subjects

At baseline, 135 patients (34 males and 101 females) agreed to participate. Among them, 106 subjects (78.5%) completed the two-year follow-up. There were no significant differences in the scores of the three scales at baseline or the demographic variables between those who attended follow-up and those who did not. Among the 106 patients (S1 Dataset), 35 patients (33.0%) were in the treated group and 71 (67.0%) were in the undertreated group. Patients in the treated group had a longer treatment duration during the two years, as compared with patients in the undertreated group (17.4 ± 6.4 *vs*. 5.0 ± 4.8 months, *p* < 0.01). Patients in the undertreated group had quit pharmacotherapy for 16.3 ± 7.4 months at the follow-up point. None of the 106 patients accepted psychotherapy at the follow-up point.

Table 1 shows the demographic variables and psychometric scores at baseline. There were no significant differences in the scores of the three scales at baseline and the demographic variables between the treated and undertreated groups (Table 1), with the exception that the SS scores were higher at baseline in the treated group as compared with the undertreated group.

**Table 1:** Demographic variables and psychometric scores (mean ± standard deviation) at baseline among the different groups.^a^

	Total (n = 106)	Treated group (n = 35)	Undertreated group (n = 71)
Age (years)	30.4 ± 8.6	32.3 ± 9.0	29.6 ± 8.3
Education (years)	13.1 ± 2.4	13.0 ± 2.6	13.1 ± 2.3
Female (%)	72.6	71.4	73.2
Married (%)	46.2	51.4	43.7
Employment (%)	51.9	48.6	53.5
HAMD	23.4 ± 4.4	23.3 ± 4.6	23.5 ± 4.3
DS	25.4 ± 5.0	26.1 ± 5.2	25.1 ± 4.9
SS	15.9 ± 6.8	18.6 ± 6.3*	14.5 ± 6.6
HADS-D	14.0 ± 3.4	13.9 ± 3.4	14.0 ± 3.4
HADS-A	15.1 ± 3.1	14.7 ± 3.1	15.3 ± 3.2

***HAMD*** = Hamilton Depression Rating Scale, ***DS*** = Depression subscale of the Depression and Somatic Symptoms Scale (DSSS), ***SS*** = Somatic subscale of the DSSS, ***HADS-D*** = Depression subscale of the Hospital Anxiety and Depression Scale (HADS), ***HADS-A*** = Anxiety subscale of the HADS

*p < 0.05

^a^ Subjects with pharmacotherapy in the index follow-up month were categorized as the treated group and those without pharmacotherapy were categorized as the undertreated group.

Among the 35 patients in the treated group, migraine was diagnosed at baseline in 19 (54.2%) patients. Among these patients, 13 (68.4%) were treated with venlafaxine (mean dosage of 126.9 ± 56.3 mg) and 6 with other antidepressants. Eight (42.1%) patients were treated with medications related to migraine prevention, such as flunarizine, propranolol, valproate, and topiramate. Among the other16 patients without migraine, 12 (75.0%) were treated with venlafaxine (mean dosage of 100.0 ± 36.9 mg) and 4 with other antidepressants. No triptan was prescribed in the 35 patients.

### Headache diagnoses and indices

Among the 106 patients at baseline, the 49 (46.2%) patients with migraine (41 migraine without aura and 8 migraine with and without aura) consisted of 44 with episodic migraine and five with chronic migraine. Among the 49 patients with migraine, 69.4% (34/49) of patients had active headache at baseline and 22.4% (11/49) patients had active headache at follow-up. Table 2 shows the case numbers of the two migraine subgroups in the treated and undertreated groups.

**Table 2:** The scores of the three scales (mean ± standard deviation) and headache indices among different subgroups at baseline and at the two-year follow-up.^c^ ^,^ ^d^

		Undertreated group			Treated group		
		No migraine	Inactive migraine	Active migraine	No migraine	Inactive migraine	Active migraine
Number		41	24	6	16	14	5
HAMD	Baseline	22.1 ± 3.9	24.4 ± 3.0	29.5 ± 5.1^**†**^	21.1±3.2	25.0±4.6	26.0±6.2
	**Follow-up**	**8.7** ± **6.5**	**10.8** ± **7.1**	**17.0** ± **8.2**	**12.6**±**6.4**	**9.1**±**5.7**	**17.0**±**6.6**
DS	Baseline	23.1 ± 4.4	27.3 ± 4.1*	29.5 ± 5.0^**†**^	24.7±3.8	27.0±6.2	28.0±6.3
	**Follow-up**	**9.4** ±± **7.6**	**10.5** ± **6.6**	**16.8** ± **10.2**	**13.9**±**7.4**	**11.1**±**5.7**	**16.8**±**9.0**
SS	Baseline	11.5 ± 5.4	17.5 ±± 5.6*	23.5 ± 5.8^**†**^	15.1±5.2	21.3±5.5*	22.4±6.3
	**Follow-up**	**6.1** ± **5.1**	**7.2** ± **5.4**	**19.7** ± **7.9** ^**†**^ ^‡^	**9.8±5.2**	**9.2±3.7**	**13.8±5.8**
HADS-D	Baseline	13.4 ± 3.3	14.6 ± 3.8	16.2 ± 1.6	13.4 ±± 2.5	13.7 ± 4.3	15.8 ± 3.3
	**Follow-up**	**5.9** ± **5.3**	**6.5** ± **4.1**	**8.3** ± **5.9**	**8.9** ± **4.3**	**7.3** ± **3.7**	**7.6** ± **3.6**
HADS-A	Baseline	14.9 ± 3.3	15.2 ± 2.8	18.7 ± 1.6^**†**^ ^‡^	13.6 ± 2.8	15.5 ± 3.1	16.2 ± 3.1
	**Follow-up**	**8.3** ± **4.5**	**8.9** ± **3.7**	**14.3** ± **4.7** ^**†**^ ^‡^	**10.0** ± **3.7**	**9.6** ± **4.2**	**11.6** ± **3.8**
Headache intensity^a^	Baseline	3.0 ±2.4	6.4 ±± 3.3*	7.7 ± 3.6^**†**^	4.9 ± 3.2	6.5 ± 2.7	7.6 ± 3.4
	**Follow-up**	**1.5** ± **2.0**	**2.1** ± **2.5**	**7.5** ± **2.2** ^**†**^ ^‡^	**2.3** ± **1.9**	**3.2** ± **2.5**	**5.8** ± **2.2** ^**†**^ ^‡^
Headache frequency^b^	Baseline	4.3 ± 6.5	10.5 ± 8.8*	15.7 ± 12.9	7.6 ± 6.1	11.4 ± 8.1	20.6 ± 13.1
	**Follow-up**	**3.0** ± **6.7**	**5.0** ± **6.6** *	**12.0** ± **10.4** ^**†**^	**4.0** ± **3.7**	**6.5** ± **5.6**	**11.2** ± **10.8**

***HAMD*** = Hamilton Depression Rating Scale, ***DS*** = Depression subscale of the Depression and Somatic Symptoms Scale (DSSS), ***SS*** = Somatic subscale of the DSSS, ***HADS-D*** = Depression subscale of the Hospital Anxiety and Depression Scale (HADS), ***HADS-A*** = Anxiety subscale of the HADS

* *p* < 0.017 for the no migraine subgroup vs. the inactive migraine subgroup.

^†^ *p* < 0.017 for the no migraine subgroup vs. the active migraine subgroup.

^‡^ *p* < 0.017 for the inactive migraine subgroup vs. the active migraine subgroup.

^a^ Headache intensity was measured by visual analog scale.

^b^ Headache frequency was calculated from the number of headache days in the past month.

^c^ Patients without migraine at baseline were categorized as the no migraine group. Patients with migraine diagnosed at baseline and with no headache or mild headache at follow-up were categorized as the inactive migraine group. Patients with migraine diagnosed at baseline and with moderate or severe headache at follow-up were categorized as the active migraine group.

^d^ Subjects with pharmacotherapy in the index follow-up month were categorized as the treated group and those without pharmacotherapy were categorized as the undertreated group.

Among the 57 patients without migraine at baseline, 13 (22.8%) had probable migraine, 28 (49.1%) had tension-type headache or probable tension-type headache, 8 (14.0%) had headache not otherwise specified or headache unspecified, and 8 (14.0%) had no headache. Among them, 22.8% (13/57) and 10.5% (6/57) patients had active headache at baseline and at follow-up, respectively.

Table 2 shows the headache intensity and frequency among the no migraine, inactive migraine, and active migraine subgroups. There was a trend that the active migraine subgroup had the greatest headache intensity and frequency, followed by the inactive migraine subgroup, both in the treated and undertreated groups. A significant difference was noted in part of the subgroups (Table 2).

Comparing headache indices at baseline and at follow-up, headache intensity and frequency exhibited significant improvement among the subgroups, except for headache frequency in the no migraine subgroup of the undertreated group and headache intensity and frequency in the active migraine subgroup both in the treated and undertreated groups.

### Differences in psychometric scores among the three subgroups

Table 2 shows the three psychometric scores. In the undertreated group, the active migraine subgroup had the highest scores on the three scales both at baseline and follow-up, followed by the inactive migraine subgroup and the no migraine subgroup. At the two-year follow-up, significant differences between subgroups were noted in the SS and HADS-A (active migraine subgroup vs. no migraine and inactive migraine subgroups).

In the treated group at baseline, a trend was noted that the active migraine subgroup had the highest scores for the three scales, followed by the inactive migraine subgroup. At the follow-up, the active migraine subgroup still had the highest scores; however, the inactive migraine subgroup had the lowest scores. At the follow-up, no significant differences were noted between subgroups in the three scales.

In the undertreated and treated groups, all the scores of the three scales in the three subgroups showed significant (*p* < 0.05) improvement at follow-up as compared with the scores at baseline, except for the HADS-A and HAMD in the active migraine subgroup of the treated group.

### The estimated differences between the two migraine subgroups vs. the no migraine subgroup

Table 3 shows the estimated differences between the two migraine subgroups vs. the no migraine subgroup at follow-up. In the undertreated group, the active migraine subgroup had significantly higher scores for depression, anxiety, and somatic symptoms, except for the HADS-D, when no migraine subgroup was used as a reference. However, the inactive migraine subgroup showed no significant differences in the three scales as compared with the no migraine subgroup.

**Table 3:** The estimate differences in scores between the no migraine group and the migraine groups at the two-year follow-up.^a^ ^.^ ^b^ ^,^ ^c^ ^,^ ^d^

	Undertreated group		Treated group	
	Inactive migraine vs. No migraine	Active migraine vs. No migraine	Inactive migraine vs. No migraine	Active migraine vs. No migraine
HAMD	2.39	8.93**	-2.59	5.60
DS	1.35	8.01*	-3.34	2.07
SS	1.04	14.61**	-0.54	3.12
HADS-D	1.20	3.70	-1.58	-1.19
HADS-A	0.64	6.06**	0.09	1.85

***HAMD*** = Hamilton Depression Rating Scale, ***DS*** = Depression subscale of the Depression and Somatic Symptoms Scale (DSSS), ***SS*** = Somatic subscale of the DSSS, ***HADS-D*** = Depression subscale of the Hospital Anxiety and Depression Scale (HADS), ***HADS-A*** = Anxiety subscale of the HADS

*p < 0.05

** *p* < 0.01

^a^ The no migraine subgroup was used as a reference.

^b^ Patients without migraine at baseline were categorized as the no migraine group. Patients with migraine diagnosed at baseline and with no headache or mild headache at follow-up were categorized as the inactive migraine group. Patients with migraine diagnosed at baseline and with moderate or severe headache at follow-up were categorized as the active migraine group.

^c^ Subjects with pharmacotherapy in the index follow-up month were categorized as the treated group and those without pharmacotherapy were categorized as the undertreated group.

^d^ The estimate difference was calculated by general linear models and represented the differences in scores between two groups after controlling for demographic variables.

In the treated group, there was no significant difference between the two migraine subgroups vs. the no migraine subgroup.

### The improvement percentage and full remission rate among the three subgroups

Table 4 shows the IPs among the three subgroups. In the undertreated group, there was a trend that the no migraine subgroup had the highest IP, followed by the inactive migraine subgroup in depression (HAMD, DS, and HADS-D) and anxiety (HADS-A). No significant differences were noted in the IPs of depression and anxiety between subgroups. In the SS, the inactive migraine subgroup had the highest IP, followed by the no migraine subgroup. A significant difference in the IP of the SS was noted between the inactive migraine subgroup vs. the active migraine subgroup. The no migraine subgroup (53.7%) also had the highest full remission rate at the follow-up point, followed by the inactive migraine subgroup (41.7%) and the active migraine subgroup (16.7%).

**Table 4:** The improvement percentages and full remission percentages among the different groups at the two-year follow-up.^a^ ^,^ ^b^ ^,^ ^c^

	Undertreated group			Treated group		
	No migraine	Inactive migraine	Active migraine	No migraine	Inactive migraine	Active migraine
HAMD (IP)	61.5	54.6	42.1	40.0	63.3	31.4
DS (IP)	60.2	59.3	44.7	42.7	55.9	39.2
SS (IP)	20.2	54.2	19.0*	13.8	52.5	37.1
HADS-D (IP)	55.3	50.8	48.1	33.5	44.3	50.2
HADS-A (IP)	43.0	39.8	24.0	22.6	36.4	27.7
Full remission (%)^d^	53.7	41.7	16.7	25.0	50.0	0
Suicide attempt (%)^e^	7.3	20.8	33.3	12.5	21.4	20.0

***IP*** = improvement percentage, ***HAMD*** = Hamilton Depression Rating Scale, ***DS*** = Depression subscale of the Depression and Somatic Symptoms Scale (DSSS), ***SS*** = Somatic subscale of the DSSS, ***HADS-D*** = Depression subscale of the Hospital Anxiety and Depression Scale (HADS), ***HADS-A*** = Anxiety subscale of the HADS

*p < 0.01 for the inactive migraine subgroup vs. the active migraine subgroup

^a^ Patients without migraine at baseline were categorized as the no migraine group. Patients with migraine diagnosed at baseline and with no headache or mild headache at follow-up were categorized as the inactive migraine group. Patients with migraine diagnosed at baseline and with moderate or severe headache at follow-up were categorized as the active migraine group.

^b^ Subjects with pharmacotherapy in the index follow-up month were categorized as the treated group and those without pharmacotherapy were categorized as the undertreated group.

^c^ The improvement percentage was calculated as (score at baseline—score at follow-up) × 100% / score at baseline.

^d^ Full remission at follow-up was defined as a HAMD score ≤ 7.

^e^ Patients made at least one suicide attempt in the past two years.

In the treated group, the inactive migraine subgroup had the highest IP in depression (HAMD and DS), anxiety (HADS-A), and somatic symptoms (SS), except for the HADS-D. No significant differences were noted in the IPs between subgroups. The inactive migraine subgroup (50.0%) had the highest full remission rate at the follow-up point, followed by the no migraine subgroup (25.0%). The active migraine subgroup had no patients with full remission.

### Duration of full remission and suicide attempt history during the two years

In the undertreated group, there was no significant difference in the percentage of full remission ≥ 2 months between patients with migraine (70.0%) and those without migraine (73.2%). The duration of full remission in patients with migraine was shorter than that in patients without migraine (6.9 ± 4.5 *vs*. 10.2 ± 6.4 months, *p* < 0.05).

In the treated group, no significant difference was noted in the percentage of full remission ≥ 2 months between patients with migraine (63.2%) and those without migraine (75.0%). In addition, the duration of full remission was not significantly different (migraine vs. no migraine: 7.8 ± 7.4 *vs*. 8.8 ± 5.8 months).

In the undertreated group, a higher percentage of patients with migraine made a suicide attempt in the past two years (23.3% *vs*. 7.3%, *p* = 0.08) as compared with patients without migraine. In the treated group, patients with migraine also tended to have a higher percentage of suicide attempts (21.1% *vs*. 12.5%, *p* = 0.67). Table 4 shows that the no migraine subgroup had the lowest rate of suicide attempts in the past two years, both in the treated and undertreated groups.

### Factors related to full remission at follow-up

Table 5 shows that active headache at the follow-up point was a significant factor related to a lower rate of full remission after controlling for demographic variables, HAMD score at baseline, migraine at baseline, and pharmacotherapy at follow-up.

**Table 5:** Factors associated with full remission of depression at follow-up.

	Wald	Odds ratio (95% CI)	*P*-value
Active headache at follow-up	6.06	0.07 (0.01–0.58)	0.01
Gender	2.88	0.39 (0.13–1.16)	0.09
Age	0.03	1.01 (0.95–1.07)	0.85
Educational years	2.16	1.17 (0.95–1.44)	0.14
Employment	0	1.01 (0.39–2.63)	0.99
Married	0.21	1.26 (0.47–3.42)	0.65
Migraine at baseline	0.13	0.84 (0.33–2.18)	0.72
HAMD scores at baseline	0.74	0.95 (0.84–1.07)	0.39
Pharmacotherapy at follow-up	0.86	0.64 (0.25–1.66)	0.35

***HAMD*** = Hamilton Depression Rating Scale

For the full sample (106 subjects), patients with active headache had a significantly lower percentage of full remission (5.9% vs. 48.3%, Odds Ratio = 0.07, 95% CI = 0.01–0.53, *p* = 0.001) as compared with patients with inactive headache.

## Discussion

Active headache at the follow-up point was a significant factor related to a lower rate of full remission at the same time point. The active migraine subgroup had the lowest rate of full remission among the three subgroups in both the treated and undertreated groups. These results demonstrated that active headache might be an important factor hindering full remission of depression. Painful physical symptoms have been reported to be a negative prognostic factor for depression [31]; however, headache has rarely been directly pointed out as an important factor in the remission of depression. Karp et al. reported that headache and muscle soreness were factors independently predicting a longer time to remission during acute treatment of recurrent depression [32]. Our result provided further evidence to support the importance of headache in the prognosis of depression. In the undertreated group at follow-up, significant differences between the active migraine subgroup *vs*. the inactive migraine and no migraine subgroups were noted in terms of somatic (SS) and anxiety (HADS-A) symptoms (Table 2). This implied that the impact of active migraine might tend to increase anxiety and somatic symptoms among patients with MDD. Migraine is related to somatosensory amplification and repeated migraine attacks might cause a similar effect to central sensitization, which is associated with comorbid pain conditions and a poorer headache-related disability [33, 34]. Migraine is an independent factor related to somatic and pain symptoms among patients with depression [19, 21]. Moreover, migraine is also related to increased anxiety symptoms [2, 11]. In fact, anxiety and somatic symptoms are common residual symptoms of depression [35, 36]. The results implied connections among active migraine, residual somatic and anxiety symptoms, and non-full remission of depression. This might be one of the possible reasons for the lowest rate of full remission in the active migraine subgroup.

In the undertreated group, the active migraine subgroup had significantly higher scores on the depression, anxiety, and somatic symptoms scales (Table 3) when the no migraine subgroup was used a reference. However, there were no significant differences between the no migraine subgroup and the inactive migraine subgroup in both the treated and undertreated groups. Previous studies have reported that MDD patients with a current MDE and migraine have greater severities of depression, anxiety, and somatic symptoms as compared with those without migraine [16, 17, 19]. The differences between patients with migraine and without migraine might be partially associated with active headache. When MDD patients were in a current MDE, most of patients with migraine might have an active headache (69.4% in this study). Therefore, MDD patients with migraine have a greater severity of somatic and anxiety symptoms [16, 17, 19]. Once the active headache improves, the gap between patients with migraine and those without migraine might decrease or disappear. This might be one possible reason for which there was no significant difference between the no migraine and inactive migraine subgroups and why migraine was not entered into the regression model. This hypothesis also explained another phenomenon, that the inactive migraine subgroup had the highest IP in the SS among the three subgroups in both the treated and undertreated groups (Table 4), and there was a significant difference in the IP of the SS between the inactive migraine subgroup and the active migraine subgroup in the undertreated group. Migraine is an independent factor related to somatic symptoms among patients with MDD [19]. Improvement of active headache in MDD patients with migraine might lead to much improvement in somatic symptoms.

There are several points worthy of note: 1) The inactive migraine subgroup in the treated group had the highest full remission rate and IP among the three subgroups. This might partially result from the fact that severe headache might exacerbate depression, anxiety, and somatic symptoms as well as hinder physical activities, and relief of active headache might lead to improvement in these symptoms simultaneously. This result implied that appropriate pharmacotherapy for migraine might be associated with a better treatment response of depression. 2) A trend was noted that both the active and inactive migraine subgroups had a higher suicide attempt rate within the past two years (Table 4). This indicates to physicians that migraine should be considered as a risk factor for suicide in clinical practice. Previous studies have also reported that subjects with migraine have an increased risk of suicide [37]. 3) In the undertreated group, patients with migraine had a shorter duration of full remission than patients without migraine. This implied that migraine might be a possible factor related to poor prognosis. However, more evidence is needed to support this hypothesis.

Several methodological issues or limitations should be addressed. 1) This was a clinical neutral study. Patients in the treated and undertreated groups were not divided by randomization, but by patients’ willingness. This might cause bias. 2) The patients were divided into several subgroups. The small sample sizes hindered statistical significance. Future studies with larger sample sizes are necessary. 3) Some of the patients without migraine had different types of headache. These patients were categorized as the no migraine subgroup because the major focus was migraine. These patients were not further divided into active and inactive headache subgroups because of the small percentage of active headache. 4) This study primarily focused on investigating the impacts of headache and migraine on patients with MDD at the follow-up point. The impacts of migraine on the longitudinal course of depression might need to be clarified.

In conclusion, active headache at follow-up is a significant factor associated with a lower rate of full remission and is related to greater severities of depression, anxiety, and somatic symptoms at follow-up among MDD patient with migraine. The active migraine subgroup in the undertreated group had greater severities of somatic and anxiety symptoms, which were related to residual symptoms of depression. Our results demonstrated that physicians should integrate the treatment plan of depression and migraine because appropriate pharmacotherapy for migraine might decrease residual symptoms and improve the full remission rate. However, these conclusions should be re-examined in future studies with larger sample sizes.

## Supporting Information

S1 Dataset The dataset includes demographic variables, headache diagnoses, headache indices, and scores of psychometric scales at baseline and 2-year follow-up among 106 patients with major depressive disorder. (XLS)
